# Cellulose Nanofiber-Based Nanocomposite Films Reinforced with Zinc Oxide Nanorods and Grapefruit Seed Extract

**DOI:** 10.3390/nano11040877

**Published:** 2021-03-30

**Authors:** Swarup Roy, Hyun Chan Kim, Pooja S. Panicker, Jong-Whan Rhim, Jaehwan Kim

**Affiliations:** 1 CRC for Nanocellulose Future Composites, Inha University, Incheon 22212, Korea; swaruproy2013@gmail.com (S.R.); kim_hyunchan@naver.com (H.C.K.); pooja.panicker7@gmail.com (P.S.P.); 2 Bio-Nanocomposite Research Center, Department of Food and Nutrition, Kyung Hee University, Seoul 02447, Korea; jwrhim@khu.ac.kr

**Keywords:** cellulose nanofiber, zinc oxide nanorod, grapefruit seed extract, nanocomposite film, antioxidant and antimicrobial activity, food packaging

## Abstract

Here, we report the fabrication and characterization of cellulose nanofiber (CNF)-based nanocomposite films reinforced with zinc oxide nanorods (ZnOs) and grapefruit seed extract (GSE). The CNF is isolated via a combination of chemical and physical methods, and the ZnO is prepared using a simple precipitation method. The ZnO and GSE are used as functional nanofillers to produce a CNF/ZnO/GSE film. Physical (morphology, chemical interactions, optical, mechanical, thermal stability, etc.) and functional (antimicrobial and antioxidant activities) film properties are tested. The incorporation of ZnO and GSE does not impact the crystalline structure, mechanical properties, or thermal stability of the CNF film. Nanocomposite films are highly transparent with improved ultraviolet blocking and vapor barrier properties. Moreover, the films exhibit effective antimicrobial and antioxidant actions. CNF/ZnO/GSE nanocomposite films with better quality and superior functional properties have many possibilities for active food packaging use.

## 1. Introduction

Plastics are widely used for packaging industrial ingredients owing to their economic efficiency, processability, and convenience. Thus, their use has rapidly increased since the 1950s at about 9% per year [1]. However, the increasing use of non-biodegradable and non-environmentally friendly petroleum-based synthetic plastics has created severe environmental problems [2,3,4]. In particular, short-term use or disposable plastics accumulate in landfills after disposal, causing enormous environmental pollution, such as soil and water pollution. They are a cause of greenhouse gas generation [5,6]. Plastics can travel as microplastics through various water, air, and soil pathways, in a manner that is dangerous to all living beings. Concerningly, recent studies have shown that humans consume about 40,000 to 50,000 microplastic particles per year [7]. Consequently, there is an urgent need for substitute materials to decrease the use of synthetic plastics. Recently, biopolymer-based nanocomposites reinforced with a low fraction of functional fillers have been considered owing to their distinctive and characteristic properties [8,9]. In this context, several environmentally friendly and biodegradable biopolymers have come to be extensively used [8,10,11]. Carbohydrate polymers have gained much attention for their excellent film-making and mechanical properties when compared to other bio-based polymers [12,13,14]. Cellulose, the most abundant biopolymer material on the planet, is an attractive carbohydrate polymer [15,16,17]. In particular, research has found the cellulose nanofiber (CNF) to have numerous applications in materials science and biomedical engineering [16,18,19,20,21]. CNF-based films have come to warrant substantial consideration in the packaging area because they are flexible, strong, environmentally friendly, and transparent [21,22,23,24,25,26,27]. The food packaging area, including active food packaging, has considerable demand for biopolymer-based films. However, low water vapor barrier properties and low functionality have limited the large-scale use of biopolymer-based films. One way to improve biopolymer films’ usefulness is to reinforce CNF films with functional fillers [25,28,29].

In this regard, natural extracts and nanofillers are ideal candidates for reinforcing CNFs to improve their physical and functional properties. In particular, zinc oxide nanorods (ZnOs) and grapefruit seed extract (GSE) are potential candidate nanofillers in CNF-based films for food packaging use. ZnO is a multifunctional nanoparticle approved by the Food and Drug Administration (FDA) and is used in several foods and cosmetics [30]. ZnO is also a very well-known nanofiller and has already been applied to fabricate numerous biopolymer-based films owing to its exceptional antimicrobial action, non-cytotoxicity, and high stability [31,32,33]. It has previously been shown that the incorporation of ZnO in biopolymers enhances their physical properties (mechanical and barrier) and provides the composite films with potent antimicrobial activities [30,34,35]. A natural functional material, GSE, has also been broadly used in food packaging applications [36,37]. Grapefruit (*Citrus paradise* Macf.) seed extract is known to have outstanding antioxidant properties and comprehensive antimicrobial activity [38]. The antioxidant property of food packaging materials is essential for active food packaging applications for nuts, fish, meats, milk, and oils. Polyphenol, tocopherol, and flavonoids are the main active compounds present in GSE [39,40]. GSE has previously been utilized to produce numerous functional films [37,38,39,41,42,43]. Various nano-ZnO contents (1–10 wt% based on biopolymer) have been used as nanofillers. However, a recent report suggested that adding 1 wt% of ZnO is enough to provide functional properties to composite films [34,35]. In GSE’s case, 5 wt% (based on a biopolymer) has been shown to be suitable for maintaining a composite film’s properties [37,44]. The enhancement of a CNF-based nanocomposite film’s properties when combined with ZnO and GSE has to date not been documented.

This study aims to fabricate CNF-based films for food packaging use by reinforcing these with ZnO and GSE. ZnO is prepared using a homogeneous precipitation technique at mild conditions deprived of surfactants. CNF/ZnO/GSE films are prepared and characterized using various analytical techniques. Furthermore, their physical and biological properties are determined. The potential for antimicrobial and antioxidant active food packaging applications of nanocomposite films is discussed.

## 2. Materials and Methods

### 2.1. Materials

Hardwood-bleached acacia elemental chlorine-free (ECF) kraft pulp was procured from Asia Pulp & Paper Co., Ltd. (Jakarta, Indonesia). Zinc acetate and potassium hydroxide were procured from DaeJung Chemicals & Metals Co., Ltd. (Siheung, Gyeonggi-do, Korea). Grapefruit seed extract (GSE, DF-100, 50% glycerol, 0.48% naringin, and other compounds) was obtained from Komipharm International Co., Ltd. (Seoul, Korea). A hardwood CNF suspension was prepared using a chemical method 2,2,6,6-Tetramethylpiperidinyloxy (TEMPO-oxidation) followed by a physical method, the so-called aqueous counter collision (ACC) method [27,45]. All other chemicals used were analytical reagent grade (purity > 99%) and were used as received.

### 2.2. Fabrication and Characterization of Zinc Oxide Nanorods

A zinc oxide nanorod (ZnO) was made using a simple precipitation method [35]. First, aqueous zinc acetate (0.1 M) solution was prepared, and then a dropwise KOH (1 M) solution was added slowly, stirred continuously, and heated at 70 °C for 2 h. After completing the reaction, the white precipitate was accumulated by filtration, before being washed with an ample amount of distilled water and then placed in an oven at 50 °C for 24 h to form dried ZnO. The ZnO’s optical properties were scrutinized using a UV-2501PC UV–Vis spectrometer (Shimadzu, Kyoto, Japan). The prepared ZnO’s morphology was characterized using field-emission scanning electron microscopy (FESEM) (SU8010, Hitachi, Matsuda, Japan). The particle size was investigated using the ImageJ program. The X-ray diffraction (XRD) pattern of the ZnO was scrutinized using an X-ray diffractometer (DMAX-2500, Rigaku, Tokyo, Japan).

### 2.3. Fabrication of CNF/ZnO/GSE Nanocomposite Films

The ZnO- and GSE-reinforced CNF-based films were made using solution mixing and the doctor blade casting technique [46]. ZnO (1.0 wt% based on CNF) and GSE (5.0 wt%) were added to the CNF water suspension and shear mixed for 20 min at 4000 rpm. Then, the suspension was sonicated (water bath) for 90 min, followed by centrifugation at 10,000 rpm for 1 h to eliminate bubbles. The film-making mixture was cast (doctor blade) on a polycarbonate plate and dried for 48 h in a cleanroom (25 °C and 45% relative humidity (RH)). The wholly dried film was removed from the plate and stored (25 °C and 45% RH) for at least 2 days before further use. A neat CNF film without any filler materials was also developed using the steps listed above. All the film specimens were made in triplicate. The prepared films were named CNF/ZnO, CNF/GSE, and CNF/ZnO/GSE, respectively, depending on the content and type of filler(s). The details of the film characterization procedures are presented in the Supporting Information. The Fourier transform infrared (FTIR) spectra of the CNF/ZnO/GSE nanocomposite films were determined using FTIR spectroscopy (Cary 630, Agilent Technol. Santa Clara, CA, USA) with a diamond crystal with a wavelength range of from 650 to 4000 cm^−1^.

## 3. Results

### 3.1. Characterization of Zinc Oxide Nanorods

**Figure 1:**
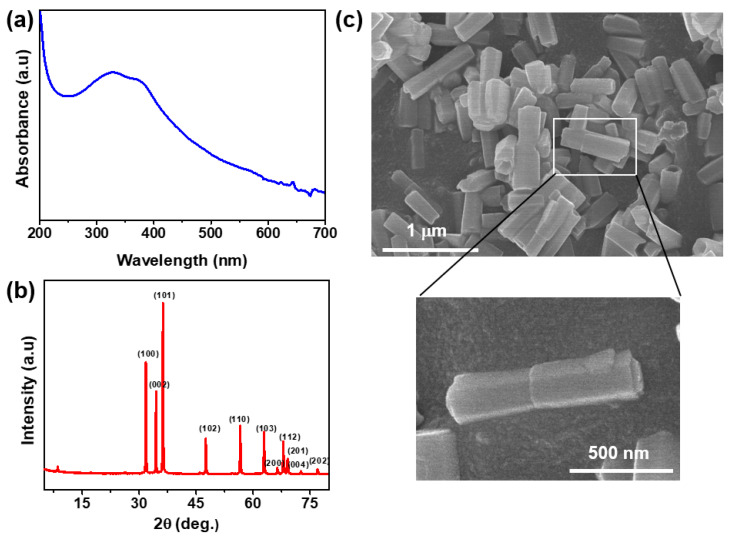
(**a**) UV–Vis spectrum, (**b**) XRD pattern, and (**c**) FESEM image of ZnO nanoparticle (NP).

The prepared ZnO was characterized by UV–Vis spectrum analysis. A peak was noticed at ~320 nm (Figure 1a), which is the characteristic absorption peak of ZnO, indicating the formation of ZnO. We analyzed it by XRD (Figure 1b) for further confirmation, and the structural analysis exhibited the unique peaks of ZnO (100, 101, 002, 102, 103, 112, 200, 201, 004, and 202). The ZnO’s obtained XRD patterns were similar to those in the previously published report, confirming ZnO synthesis [35]. As for morphology, we further characterized ZnO by FESEM (Figure 1c); the prepared material was rod-shaped in a size range of 280–500 nm with an average length of 406 ± 54 nm and an average diameter of 140  ±  29 nm. The prepared ZnO’s morphology was thus comparable to previously collected data [35].

### 3.2. CNF/ZnO/GSE Nanocomposite Films

#### 3.2.1. Morphologies

**Figure 2:**
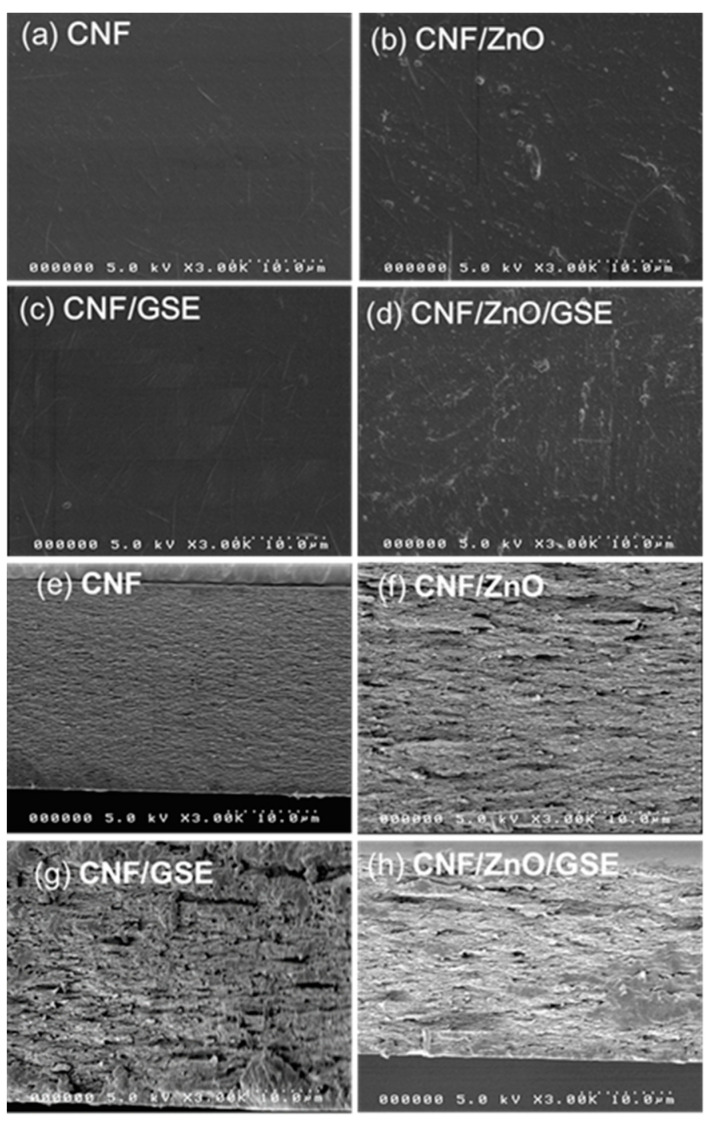
FESEM surface (**a**–**d**) and cross-sectional (**e**–**h**) images of the neat cellulose nanofiber (CNF) and CNF/ZnO/grapefruit seed extract (GSE) nanocomposite films.

For nanocomposite film preparation, the fillers’ uniform dispersion in a biopolymer matrix is essential. To this end, the film’s microstructure was investigated using FESEM, and the outcomes are shown in Figure 2. The FESEM images (surface and cross-sectional) of the neat CNF and its composite films were analyzed. The surface images (Figure 2a–d) displayed how all of the films were unbroken, free of cavities or cracks. ZnO and GSE were uniformly distributed within the CNF matrix, with no significant aggregation of fillers. Cross-sectional views (Figure 2e–h) show that the neat CNF and its nanocomposite films had a layered structure, indicating excellent compatibility and miscibility between the CNF matrix and the filler materials. In a melanin nanoparticle (MNP)-incorporated CNF film, similar results were observed previously [27]. 

**Figure 3:**
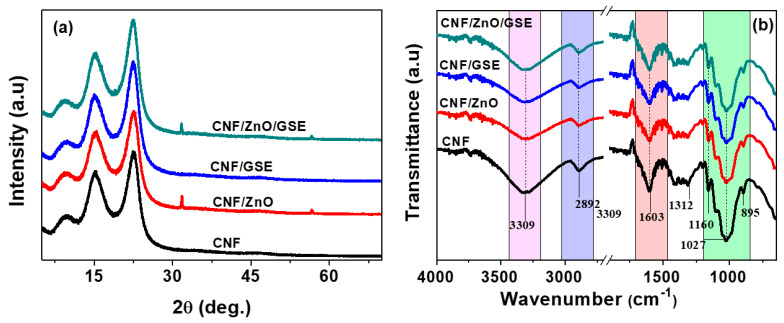
(**a**) XRD patterns and (**b**) FTIR spectra of the neat CNF and CNF/ZnO/GSE nanocomposite films.

The XRD patterns of all of the films are displayed in Figure 3a. The neat CNF showed two prominent peaks, at 2θ = 15.1° and 22.5°, matching the (110) and (002) planes of cellulose I, respectively [47]. The obtained data indicated that the crystalline parts were preserved in the nanofiber. The nanocomposite films’ XRD patterns, likewise, displayed comparable peaks, demonstrating that by mixing fillers in the CNF matrix, the structure of cellulose remained unaltered. In a ZnO-added film, few characteristic diffraction peaks were seen, indicating ZnO’s presence in the film. The crystallinity index (CI) values were determined using Equation (S1) in Supplementary Materials and calculated as 59.21%, 59.31%, 61.26%, and 60.45% for CNF, CNF/ZnO, CNF/GSE, and CNF/ZnO/GSE, respectively. ZnO and GSE alone, and their combination with CNF, did not change the CI, meaning the nanocomposite films showed a similar crystal structure to the neat CNF film. Previously, similar results were observed in MNP-reinforced CNF films [27].

#### 3.2.2. FTIR and Optical Properties

The FTIR spectra of all of the films are displayed in Figure 3b. A broad peak at ~3309 cm^−1^ was noticed in all films, referred to as the O-H stretching vibration (cellulose) [27]. The peak found at ~2892 cm^−1^ occurred due to the cellulose chain’s alkane groups (C-H stretching vibrations). The peak found at 1603 cm^−1^ was owing to the –COO stretching (TEMPO) [48]. The peak at 1312 cm^−1^ was designated to the CH_2_ wagging (cellulose) [49]. Peaks detected at 1160 and 1027 cm^−1^ matched the C-C and C-O-C pyranose stretching (cellulose), respectively [50]. The peak at 895 cm^−1^ was related to cellulosic β-glycosidic linkage [49]. In the nanocomposite films, most peaks were like those of the neat CNF apart from slight modifications in their intensities. The data showed that the functional groups of the CNF-based films were not significantly altered, reinforcing the notion that the incorporation of fillers did not affect the chemical structure. The slight distinctions in peak intensities were probably due to van der Walls interactions among ZnO, GSE, and CNF [34].

**Figure 4:**
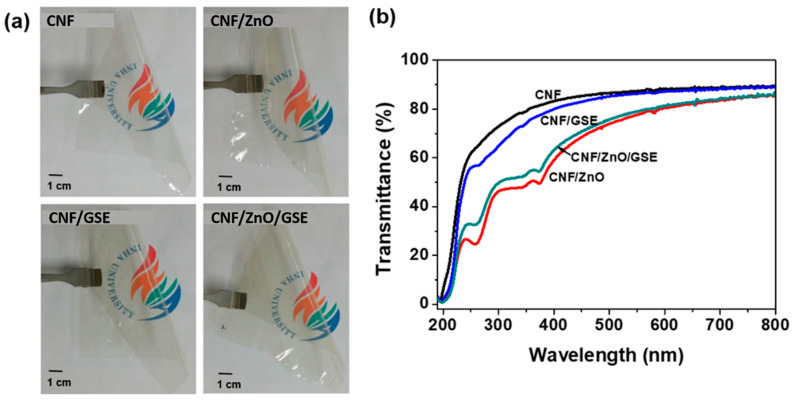
(**a**) The visual appearance photos and (**b**) UV–Vis spectra of CNF/ZnO/GSE films.

Nanocomposite films’ visual appearance and optical properties are crucial for packaging. The macroscopic visual appearances of the neat CNF and its nanocomposite films are presented in Figure 4a. All of the prepared films were highly flexible and see-through. The neat CNF film was colorless, whereas the nanocomposite films were light yellowish. The color of the nanocomposite film is primarily reliant on the nature of the filler’s material. The UV–Vis spectra of all of the films are illustrated in Figure 4b. The neat CNF film showed low light absorption in both UV and visible light.

In contrast, the nanocomposite films with ZnO showed improved absorption (UV region) due to ZnO’s light absorption. The blending of GSE in CNF did not affect the optical properties of the CNF film. The UV-blocking and transparency properties of all of the films were characterized using the percentage transmittance at 280 and 660 nm (Table 1). The UV-blocking (T_280_) property of the ZnO- and GSE-added CNF films was inferior to that of the neat CNF film. For the CNF/ZnO film, the T_280_ was almost half that of the neat CNF film, whereas, in CNF/GSE, it was not much decreased. The transparency (T_660_) of the controlled CNF film was 89.1%; in contrast, the CNF/ZnO, CNF/GSE, and CNF/ZnO/GSE nanocomposite films had transparencies of 81.6%, 87.5 %, and 84.3%, respectively. A GSE-incorporated gelatin film showed that the addition of GSE does not sacrifice the film [38]. Amjadi et al. [30] also showed that adding ZnO to a gelatin-based film does not influence the film’s whiteness indices. Such insight supports the finding that the prepared nanocomposite films were highly transparent, with more than 80% transparency. Furthermore, the developed CNF-based nanocomposite films showed a slightly improved UV-blocking effect, which could be useful for active packing applications.

#### 3.2.3. Moisture Content (MC), Swelling Ratio (SR), and Water Vapor Permeability (WVP)

**Figure 5:**
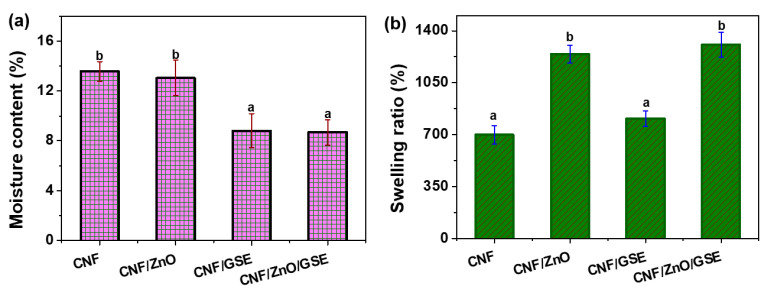
Moisture content (**a**) and swelling ratio (**b**) of the CNF/ZnO/GSE films. Data with the same superscript letter within the same column are not significantly (*p* > 0.05) different from Duncan’s multiple range tests.

The SR and MC of all of the films are presented in Figure 5. The MC of the neat CNF film was 13.6 ± 0.8%, which decreased after incorporating ZnO and GSE in the CNF. The decrease was not significant for ZnO alone, whereas, for GSE and ZnO/GSE, the changes were significant compared to the neat CNF. The films’ MC dropped due to the hydrophilicity reduction associated with the incorporation of filler materials; consequently, the accessibility of the free -OH group decreased. For an MNP-incorporated CNF film, analogous results were detected earlier [27].

The neat CNF film’s SR was 698.4 ± 62%, indicating a swellable composite with excellent water holding capacity. The nanocomposite film’s SR was significantly improved in fillers’ presence, depending on the type of filler. In ZnO’s case, the swelling increase was high when compared to GSE, whereas, for ZnO/GSE, the SR was comparable to ZNO alone in the CNF. The obtained results indicate that the raised SR of the films is mainly related to ZnO. The SR of biopolymer-based films relies mainly on the materials’ intrinsic properties (crosslinking, porosity, etc.). In the present case, nanocomposite films’ raised SRs might be due to increased crosslinking density and increments in porosity [50].

The WVP values of all of the films are shown in Table 1. The WVP of the neat CNF film was 0.55 ± 0.02 × 10^−9^ g·m/m^2^·Pa·s, which was reduced after incorporating ZnO, whereas it did not change for GSE. In the ZnO-added CNF film, the WVP was 0.46 ± 0.01 × 10^−9^ g·m/m^2^·Pa·s, whereas, for the ZnO/GSE, it was 0.51 ± 0.01 × 10^−9^ g·m/m^2^·Pa·s. The decreased WVP of the CNF-based film was possibly owing to the formation of a tortuous lane to vapor produced by well-dispersed ZnO in the polymer matrix [27]. Previously, ZnO addition in a carrageenan film enhanced the vapor barrier property [35]. In contrast, GSE incorporation did not meaningfully alter the water vapor barrier property of an agar-based film [38].

#### 3.2.4. Mechanical Properties

**Figure 6:**
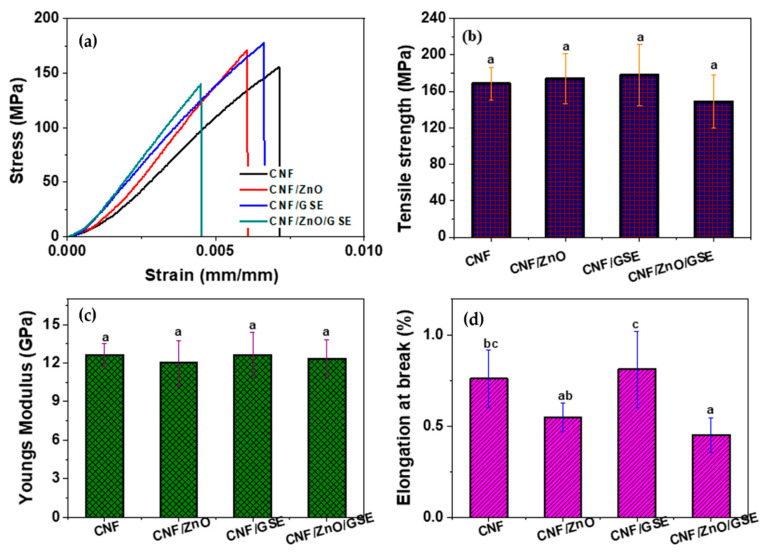
Mechanical properties of CNF/ZnO/GSE films: (**a**) stress–strain curves, (**b**) tensile strength, (**c**) Young’s modulus, (**d**) elongation at break. Data with the same superscript letter within the same column are not significantly (*p* > 0.05) different from Duncan’s multiple range tests.

The stress–strain curve, tensile strength (TS), Young’s modulus (YM), and elongation at break (EB) for each film are shown in Figure 6. The average thickness of the CNF-based films was 16 ± 3 μm. Figure 6 shows that the films’ mechanical properties were not significantly changed by reinforcing a CNF with ZnO/GSE. The TS and YM of the nanocomposite film were like those of the neat CNF film, while the EB was slightly changed. The results indicate no reduction in the CNF film’s properties after incorporating fillers. In the case of a ZnO-included carrageenan-based film, it was reported that the ZnO blending did not impact the film’s mechanical behaviors [35].

In contrast to the present observation, for a ZnO-added gelatin-based film, a substantial enhancement in mechanical properties was reported previously [30]. The addition of a low GSE content does not much affect the mechanical behaviors of a biopolymer-based film, as was previously shown in carrageenan/GSE composite films [41]. The GSE in the CNF slightly increased the EB, which it is reasonable to think occurred due to glycerol (plasticizer) in the GSE. In a GSE-added chitosan composite film, an increase in the EB was observed [42]. Biopolymer-based films’ mechanical properties mostly depend on nanofillers’ distribution and their interactions with polymers [51].

#### 3.2.5. Thermal Analysis

**Figure 7:**
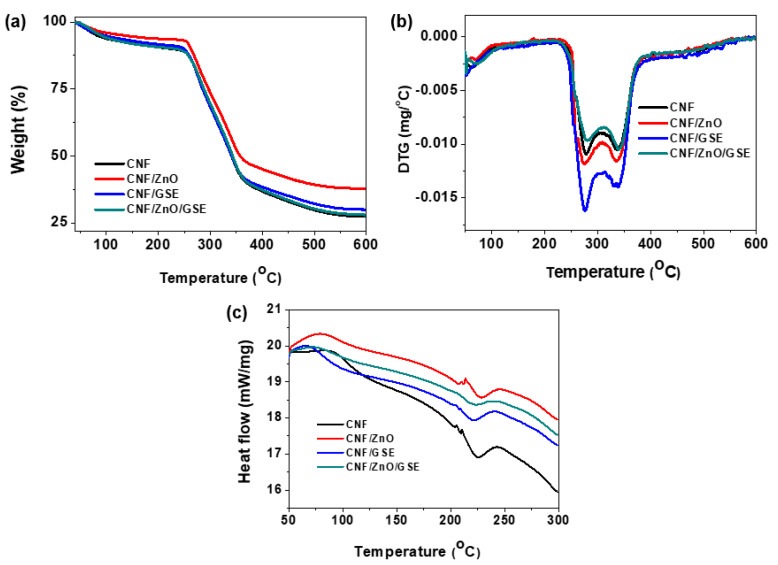
Thermal analysis of CNF/ZnO/GSE films: (**a**) TGA, (**b**) DTG plots, and (**c**) differential scanning calorimetry (DSC).

The thermogravimetric analysis (TGA) and derivative thermogravimetry (DTG) results of all of the films are presented in Figure 7. All of the developed films showed two-stage thermal decomposition. Moreover, another degradation took place at the ~65 °C for the neat CNF and ~70–75 °C for the composite films, associated with the moisture’s vaporization. The first maximum weight loss occurred at around ~275–280 °C due to the cellulose’s thermal cleavage of glycosidic linkages’ breakdown. The maximum weight loss was detected in the final step at ~340 °C for all of the tested films, typically due to cellulose [27,52]. The detailed results of the thermogravimetric analysis are presented in Table 2. The T_onset_/T_end_ temperature was almost the same for all of the tested films.

In contrast, T_5%_ (5% decomposition) varied depending on the filler’s composition. The decomposition temperature of the nanocomposite films was more comparable to that of the neat CNF film. The T_50%_ (half degradation) of all samples showed the highest temperature for the ZnO-added case, whereas the other cases were similar. This observation reveals that neither GSE nor ZnO/GSE meaningfully affected the thermal stability of the CNF—only ZnO enhanced the thermal stability of the CNF-based nanocomposite films. ZnO’s blending in a carrageenan-based film improved the composite film’s thermal stability [35]. It has also been described that the blending of GSE in a carrageenan film did not pointedly alter the thermal stability of the composite film [41]. The final char matter values of the CNF, CNF/ZnO, CNF/GSE, and CNF/ZnO/GSE films at 600 °C were 26.5, 37.1, 29.6, and 26.8%, respectively, indicating high char content (<25%), which is due to the existence of a non-flammable mineral in the cellulose material. ZnO’s thermal stability may cause a higher char content than that of the neat CNF [35]. 

Differential scanning calorimetry (DSC) analysis was also carried out to ascertain the nanocomposites’ formations (see Figure 7c). The small peak detected in the range of 70 to 110 °C is related to the endothermic peak that appeared due to a loss of water vapor [27,52]. In CNF-based nanocomposite films, significant shifting of the first peak was detected, which might be due to the inconstant water evaporation. The ZnO-added nanocomposite film’s final exothermic peak slightly increased, whereas for the GSE- and ZnO/GSE-incorporated films, a slight decrease was noticed. Overall, the present observation suggests a minor variation in thermal properties due to the fillers’ blending, which is compatible with the CNF.

#### 3.2.6. Antimicrobial Activity

**Figure 8:**
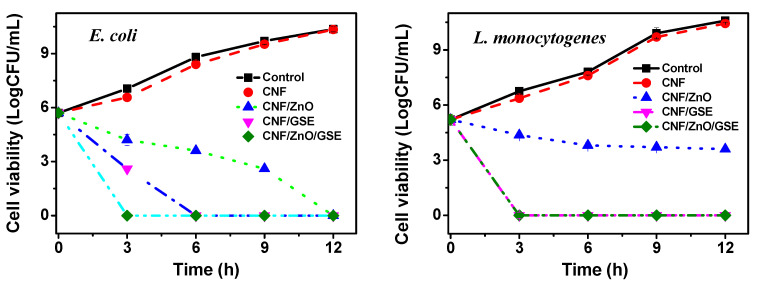
Antimicrobial activity of CNF/ZnO/GSE films.

The antimicrobial activity of the CNF-based films is displayed in Figure 8. The neat CNF film exhibited somewhat less growth (both test bacteria) compared to the control. In contrast, the films incorporated with ZnO and/or GSE presented substantial antibacterial activity. The ZnO-blended film presented slow antibacterial activity and resisted the complete growth of *Escherichia coli* only after 12 h, as lower content (1%) was used in this study. The ZnO-blended film presented low antibacterial activity against *Listeria monocytogenes*, displaying continuous bacterial growth delay due to ZnO’s antimicrobial function. The antibacterial action of ZnO was more in *E. coli* compared to *L. monocytogenes*, which is most likely due to their cell wall structure difference. The antimicrobial activity of nano-ZnO is identified as dependent on size, shape, and synthesis conditions. It is believed that the Zn^2+^ ions increase membrane permeability by disordering the bacterial cell membrane. In this process, many reactive oxygen species are also generated, which leads to oxidative stress and consequently cell death [34,53]. ZnO’s intense antibacterial activity, when added to an active packaging film, had been already discussed in several published research papers [54,55,56]. 

The GSE-incorporated films alone and GSE/ZnO-added films showed potent antibacterial activity against both test bacteria, although they were more effective against *L. monocytogenes* than *E. coli*. The results indicate that GSE is more active in inhibiting Gram-positive bacterial growth than Gram-negative bacteria because of the structural alteration. GSE contains many polyphenol compounds (such as naringin, limonin, etc.) known to have an antimicrobial function [57]. GSE enters the cell by rupturing the membrane and may attach to cellular proteins to disable their function [37,39]. Previously, in several reports, the vigorous antibacterial activity of GSE has been reported [37,44,57,58,59]. A CNF-based nanocomposite film with intense antibacterial action can help a packaging film to expand a food’s shelf life by preventing microbial contamination.

#### 3.2.7. Antioxidant Activity 

**Figure 9:**
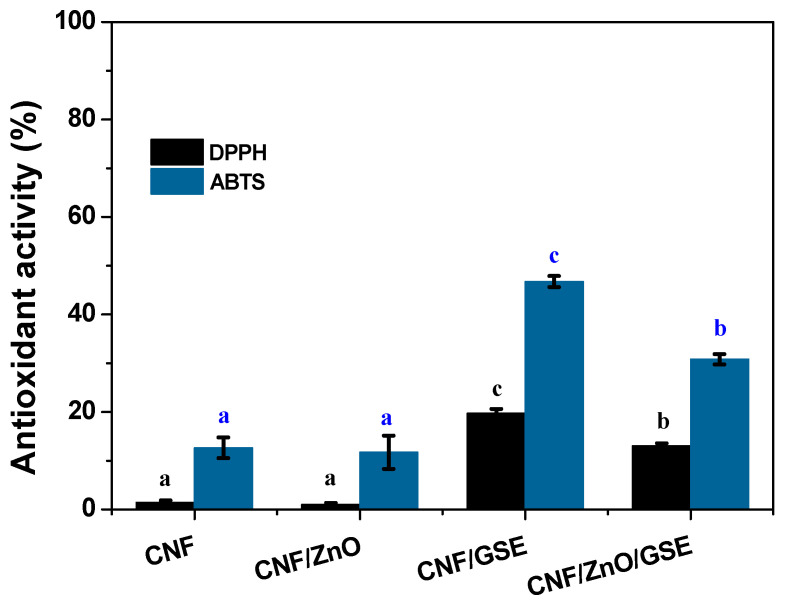
Antioxidant activity of CNF/ZnO/GSE films. Data with the same superscript letter within the same column are not significantly (*p* > 0.05) different from Duncan’s multiple range tests.

Figure 9 shows the antioxidant activities of all of the films. The 2,2-diphenyl-1-picrylhydrazyl (DPPH) radical scavenging efficiency of the control film and ZnO-added film was negligible, whereas the GSE-added film exhibited enhanced antioxidant activity. Similar to the DPPH results, the 2,2′-azino-bis(3-ethylbenzothiazoline-6-sulfonic acid) (ABTS) radical scavenging efficiency of the ZnO-added film was identical to that of the neat CNF film but significantly improved for the CNF/GSE and CNF/ZnO/GSE films. GSE and ZnO’s combined use showed a lesser effect due to the antioxidant-inactive ZnO. Similar results were reported previously in the ZnO case and for a curcumin-added CMC-based film [34]. Nanocomposite films’ increased antioxidant activity is mainly due to a potent antioxidant, which comes primarily from the tocopherol, ascorbic acids, and other flavonoids in GSE [38]. The excellent antioxidant activity of GSE glyceric extract has also been reported [60]. It is readily established that biopolymer-based films’ antioxidant activity is linearly proportional to antioxidant material [61]. Previously, the excellent antioxidant activity of GSE in vegetable oil was reported [40]. Further to this, a GSE-added poly(vinyl alcohol)-based film’s intense antioxidant activity was reported recently [44]. Prepared CNF-based films with enhanced antioxidant activity are suitable for food packaging applications. The antioxidant nanocomposite film can also be expected to shield oxidation-prone foods and extend the foods’ shelf life.

## 4. Conclusions

We prepared CNF-based films by incorporating ZnO and GSE as functional nanofillers. The ZnO and GSE were suited to cellulose and unvaryingly dispersed in the polymer matrix. The reinforcement of functional fillers in CNF-based nanocomposite films improved their UV-light blocking and water vapor barrier properties. In contrast, the nanocomposite films’ crystallinity, mechanical properties, and thermal stability were not meaningfully affected. All of the prepared nanocomposite films showed highly remarkable transparency. The CNF-based nanocomposite film also exhibited intense antimicrobial activity against food-borne pathogens and good antioxidant activity. The highly transparent CNF/ZnO/GSE films with better physical and functional properties (antimicrobial and antioxidant) have excellent potential to be used as active food packaging materials.

## Figures and Tables

**Table 1:** Light transmittance and water vapor permeability of CNF/ZnO/GSE films *.

Films	T_280_ (%)	T_660_ (%)	WVP (×10^−9^ g·m/m^2^·Pa·s)
CNF	68.89 ± 0.30 ^d^	89.05 ± 0.93 ^c^	0.55 ± 0.02 ^b^
CNF/ZnO	35.23 ± 1.98 ^a^	81.57 ± 1.51 ^a^	0.46 ± 0.01 ^a^
CNF/GSE	57.75 ± 0.82 ^c^	87.48 ± 0.30 ^c^	0.56 ± 0.05 ^b^
CNF/ZnO/GSE	44.75 ± 2.14 ^b^	84.32 ± 0.79 ^b^	0.51 ± 0.01 ^ab^

* Data with the same superscript letter within the same column are not significantly (*p* > 0.05) different from Duncan’s multiple range tests.

**Table 2:** Thermogravimetric analysis data of CNF/ZnO/GSE nanocomposite films.

Films	T_onset_/T_end_ (°C)	T5% (°C)	T50% (°C)	Char Content (%)
CNF	220/390	83	341	26.5
CNF/ZnO	228/392	135	357	37.1
CNF/GSE	222/389	96	341	29.6
CNF/ZnO/GSE	221/388	90	343	26.8

## Supplementary Materials

Film characterization methods are available online at https://www.mdpi.com/article/10.3390/nano11040877/s1.
