# Supplementary material for: Cellulose Nanofiber-Based Nanocomposite Films Reinforced with Zinc Oxide Nanorods and Grapefruit Seed Extract
Source: Nanomaterials (Basel). 2021 Mar 30;11(4):877. doi: 10.3390/nano11040877 (PMC8066394; doi:10.3390/nano11040877)
Supplement: Supplementary file 1 [file nanomaterials-11-00877-s001.pdf]

nanomaterials

MDPI

Supplementary material:

Cellulose Nanofiber-Based Nanocomposite Films Reinforced with Zinc Oxide Nanorod and Grapefruit Seed Extract

Swarup Roy [1,2], Hyun Chan Kim [1], Pooja S. Panicker [1], Jong-Whan Rhim [2], Jaehwan Kim [1,*]

[1] CRC for Nanocellulose Future Composites, Inha University, Incheon 22212, Korea; swaruproy2013@gmail.com (S.R.); kim_hyunchan@naver.com (H.C.K.); pooja.panicker7@gmail.com (P.S.P)
[2] Department of Food and Nutrition, BioNanocomposite Research Center, Kyung Hee University, 26 Kyungheedae-ro, Dongdaemun-gu, Seoul 02447, Korea; jwrhim@khu.ac.kr
* Correspondence: jaehwan@inha.ac.kr; Tel.: +82-32-874-7325

Film Characterization Methods

Morphology and Structure

The surface and cross-sectional morphologies of the pristine CNF and CNF/ZnO/GSE nanocomposite films were scrutinized using a FESEM (S4000, Hitachi, Tokyo, Japan) at an accelerating voltage of 5 kV. All the film specimens were sputter-coated with platinum for 90 s before the measurement. XRD patterns of the samples were examined using a multi-purpose X-ray Diffractometer (DMAX-2500, Rigaku, Tokyo, Japan) with Cu Kα radiation incident X-ray source (40 kV, 300 mA) over the range of 2θ = 5 to 80°. The crystallinity index (CI) was determined with the help of the following equation:

$$\mathrm{CI}\ (\%) = \frac{I_{002} - I_{am}}{I_{002}} \times 100 \qquad \text{(S1)}$$

Where I_{002} is the intensity at (002) peak, and I_{am} is the intensity at the minimum between (110) and (002) peaks [1].

FTIR and Optical Properties

FTIR spectra were noted using an FTIR spectrometer (Bruker VERTEX 80V, Billerica, MA, USA) in attenuated total reflection (ATR) mode with the wavenumber range of 4000-650 cm^{-1}, and 16 scan rates with 4 cm^{-1} resolution. The optical properties were measured using a UV-vis spectrophotometer (UV-2501PC, Shimadzu, Kyoto, Japan) in 190–800 nm. The films' UV-barrier property and transparency were also evaluated by measuring the light transmittance at 280 nm (T_{280}) and 660 nm (T_{660}), respectively [2].

Moisture Content, Swelling Ratio, and Water Vapor Permeability

The moisture content (MC) of the nanocomposite film was determined according to the established method [3]. The MC of the nanocomposite films (2.5 cm × 2.5 cm) was calculated as the film's weight change after drying at 105 °C for 24 h using the following equation:

$$\mathrm{MC}\ (\%) = \frac{W_1 - W_2}{W_1} \times 100 \qquad \text{(S2)}$$

where W_1 and W_2 refer to the initial and dried weight of the film samples, respectively.

For measuring the swelling ratio (SR) of film, a pre-weighed film sample (2.5 cm × 2.5 cm) was submerged in 20 mL DI water for 1 h and took out from the water and weighed after removing surface water using a blotting paper. The SR of the films was

$$\mathrm{SR}\ (\%) = \frac{W_2 - W_1}{W_1} \times 100 \quad \text{(S3)}$$

where W_1 and W_2 refer to the initial and final weight of the film samples correspondingly.

Water vapor permeability (WVP) of the CNF/ZnO/GSE nanocomposite films was determined using a WVP cup by following the ASTM E96-95 standard method. WVP (g.m/m^2.Pa.s) of the nanocomposite films was determined as follows:

$$\mathrm{WVP} = \frac{\Delta W \times L}{t \times A \times \Delta P} \quad \text{(S4)}$$

where ΔW is the weight alteration of the WVP cup (g), L is the thickness of the film (m), ΔP is the partial water vapor pressure difference across the two sides of the film, A is the permeation area of the film (m^2), and t is the time (s) [4].

Mechanical Properties

The film samples' thickness was measured using a digital micrometer (Digimatic Micrometer, QuantuMike IP 65, Mitutoyo, Japan) with 1 m accuracy. Mechanical properties of the prepared nanocomposite films were tested according to the standard (ASTM D-882-97) with a household tensile testing system (SSCW 120, Won Shift Precision, Hwaseong, South Korea) [5]. The nanocomposite films were cut into dimensions of 5 cm × 1 cm to estimate tensile strength (TS), Young's modulus (YM), and elongation at break (EB). The gauge length and applied pulling rate was 20 mm and 0.005 mm/s, respectively. The tensile test was performed in a controlled environment at 25 °C and 30% RH. Four samples were tested for each case and averaged the values.

Differential Scanning Calorimetry and Thermogravimetric Analysis

Differential scanning calorimetry (DSC) was examined using a TA instrument (DSC200 F3, Netzsch, Selb, Germany) at a heating rate of 10 °C/min between 20 and 300 °C under a nitrogen gas atmosphere. Thermogravimetric analysis (TGA) measurements were carried out using a TGA (STA 409 PC, Netzsch, Selb, Germany), and ~ 10 mg film samples were tested at a heating rate 10 °C/min in a temperature range of 30 to 600 °C under a nitrogen flow of 20 cm^3/min. The maximum disintegration temperature was determined from a derivative form of TGA (DTG) curves [6].

Antimicrobial Activity and Antioxidant Activity

The films' antibacterial activity was studied using food-borne pathogenic bacteria, *L. monocytogenes,* and *E. coli* using a total viable colony count (TVCC) method [7]. For this, *L. monocytogenes* and *E. coli* were aseptically inoculated in the BHI and TSB broth, respectively, and then cultured overnight at 37 °C with agitation at 100 rpm. The inoculum was then diluted (~10^6 CFU/mL), transferred aseptically into a 100 mL conical tube containing 20 mL of culture broth with 100 mg of film sample incubated for 12 h at 37 °C under mild agitation. Samples were taken at predetermined time intervals, each sample was serially diluted and plated on an agar plate, and viable cells were counted. All tests were performed in triplicate, and the average value was reported.

The films' antioxidant activities were determined by assessing the free radical scavenging activity using DPPH and ABTS radical scavenging method [8,9]. For DPPH

analysis, a prescribed amount of methanolic solution of DPPH was freshly made, and ~50 mg of tested film sample was added in a 5 mL DPPH solution and incubated at room temperature for 30 min and measured the absorbance at 517 nm. At first, in the ABTS assay, potassium sulfate was added to the ABTS solution and incubated overnight to make the ABTS assay solution. In a 5 mL of ABTS assay solution, ~50 mg of the film samples were mixed and incubated at room temperature, and then absorbance measured at 734 nm. A control was also tested without adding the film sample in both assay solution. The antioxidant activity of the CNF/ZnO/GSE nanocomposite films was determined as follows:

$$\text{Free radical scavenging activity (\%)} = \frac{A_c - A_t}{A_c} \times 100 \qquad \text{(S5)}$$

where A_c and A_t was the absorbance of DPPH/ABTS of the control and test film, respectively.

Statistical Analysis

The films' properties were measured in triplicate with individually prepared films as replicated experimental units. One-way analysis of variance (ANOVA) was performed, and the significance of each mean property value was determined ($p < 0.05$) by Duncan's multiple range test using the SPSS statistical analysis computer program for Windows (SPSS Inc., Chicago, IL, USA).

References

1. Jayaramudu, T.; Varaprasad, K.; Kim, H.C.; Kafy, A.; Kim, J.W.; Kim, J. Calcinated tea and cellulose composite films and its dielectric and lead adsorption properties. *Carbohyd. Polym.* **2017**, *171*, 183–192, doi:10.1016/j.carbpol.2017.04.077.
2. Roy, S.; Rhim, J.-W. Carrageenan-based antimicrobial bionanocomposite films incorporated with ZnO nanoparticles stabilized by melanin. *Food Hydrocoll.* **2019**, *90*, 500–507, doi:10.1016/j.foodhyd.2018.12.056.
3. Roy, S.; Rhim, J.-W.; Jaiswal, L. Bioactive agar-based functional composite film incorporated with copper sulfide nanoparticles. *Food Hydrocoll.* **2019**, *93*, 156–166, doi:10.1016/j.foodhyd.2019.02.034.
4. Roy, S.; Rhim, J.-W. Melanin-mediated synthesis of copper oxide nanoparticles and preparation of functional agar/CuO NP nanocomposite films. *J. Nanomater.* **2019**, *2019*, doi:10.1155/2019/2840517.
5. Roy, S.; Kim, H.C.; Zhai, L.; Kim, J. Preparation and characterization of synthetic melanin-like nanoparticles reinforced chitosan nanocomposite films. *Carbohydr. Polym.* **2020**, *231*, 115729, doi:10.1016/j.carbpol.2019.115729.
6. Roy, S.; Shankar, S.; Rhim, J.-W. Melanin-mediated synthesis of silver nanoparticle and its use for the preparation of carrageenan-based antibacterial films. *Food Hydrocoll.* **2019**, *88*, 237–246, doi:10.1016/j.foodhyd.2018.10.013.
7. Roy, S.; Rhim, J.-W. Antioxidant and antimicrobial poly (vinyl alcohol)-based films incorporated with grapefruit seed extract and curcumin. *J. Environ. Chem. Eng.* **2020**, 10.1016/j.jece.2020.104694, 104694, doi:10.1016/j.jece.2020.104694.
8. Roy, S.; Rhim, J.-W. Preparation of antimicrobial and antioxidant gelatin/curcumin composite films for active food packaging application. *Colloids Sur. B. Biointerf.* **2020**, *188*, 110761, doi:10.1016/j.colsurfb.2019.110761.
9. Roy, S.; Rhim, J.-W. Curcumin incorporated poly (butylene adipate-co-terephthalate) film with improved water vapor barrier and antioxidant properties. *Materials* **2020**, *13*, 4369, doi:10.3390/ma13194369.
